# Editorial: Nutrition and sustainable development goal 10: reduced inequalities

**DOI:** 10.3389/fnut.2025.1557562

**Published:** 2025-01-29

**Authors:** Emmanuel Cohen

**Affiliations:** Eco-Anthropology (EA UMR 7206), Centre National de la Recherche Scientifique (CNRS), National Museum of Natural History, Paris, France

**Keywords:** nutrition, sustainable development, determinants of health, agro-food systems, urbanization

The United Nations Sustainable Development Goals (SDGs) are the cornerstone of the 2030 Agenda for Sustainable Development. Nutrition-related challenges occupy an important place within this agenda. Indeed, the increasing prevalence of cardiometabolic diseases in the last decades constitutes the main concern for public health worldwide and it can have a lasting impact on the development of urbanized societies (1). Moreover, the necessity to develop sustainable and resilient agro-food systems to protect the environment has become a priority in all international policies (2). Accordingly, in a globalized world experiencing an overall urban transition, the Nutrition and Sustainable Development Goal (SDG) 10: Reduced Inequalities, aims to provide new theoretical and empirical insights, on nutrition-related issues situated at the crossroads of health and environmental dimensions, to identify at-risk subgroups in different socio-ecological contexts and to suggest alternatives to improve both human and environmental health.

Through the articulation of 14 relevant contributions that include 10 original case studies conducted worldwide, 1 clinical trial, 2 literature reviews, and 1 brief research report, the SDG 10: Reduced Inequalities provided relevant outcomes along with critical analyses and interpretations to illustrate challenges and initiatives around nutrition-related health and environmental issues. Through contributions focusing on the determinants of nutritional health, but also on issues and orientations of agro-food systems at both the national and regional levels, the SDG 10: Reduced Inequalities provides an original overview of factors leading to at-risk dietary patterns and potential socio-political strategies to address such public health issues in a medium- or long-term perspective. This Research Topic has a specific focus on Low- and Middle-income Countries where the urbanization rates are highest (3), as the incidence of obesity-related cardiometabolic diseases (4), while the sustainability of agro-food systems becomes a social priority with increasing soil deterioration (5).

Hence, the first two contributions, by Shifera et al. and Mengstie et al., focused on the determinants of malnutrition in Ethiopia. These works showed that rural and socially excluded populations are more exposed to undernutrition, both for children and mothers. Such trends are in line with the literature, which highlights that countries in the early stages of urban transition present persistently high rates of stunting, wasting and underweight. In continuity with these works, two contributions in Iran, by Ebrahimi et al. and Roustaee et al., showed that along the urbanization process, the consumption of processed energy-dense foods increases in multiple age groups, with a higher risk of obesity-related cardiometabolic diseases. Such a comparative framework sheds light on how countries in more advanced stages of urban transition, such as Iran, are more exposed to new forms of malnutrition like over nutrition, compared to countries that still maintain a pre-industrial lifestyle in some spaces, such as Ethiopia or Bangladesh where another relevant contribution was realized by Islam et al.. Then, Mhamad et al. showed that the prevalence of stunting among preschool children in Iraq is relatively low in some urban areas of the country. The SDG 10 also benefits from a scoping review conducted by John et al. in Nigeria, the largest economy in Africa, which highlights the evidence of an ongoing double burden of malnutrition among children under five years, between persisting undernutrition favoring wasting/stunting and increasing over nutrition favoring overweight/obesity. Meanwhile, in a high income country like China, Jiang et al. described how the social isolation of the elderly reduces health-related quality of life.

This Research Topic presents several contributions on socio-political strategies to improve nutritional health, especially in urbanized areas, with potential positive fallouts on environmental health. For example, in addition to the previous contribution from Nigeria by John et al., which recommends health policy interventions at all levels (individual, household, regional and national), Stadlmayr et al. conducted a scoping review in sub-Saharan Africa highlighting the necessity to improve the food environment to favor greater access to fruits and vegetables, through food safety, sustainable local production, lower prices and food diversity. Another contribution by Hudson et al. showed that school gardening, nutrition education, and cooking programs delivered to elementary children may positively influence the home food environment in the Austin area of Texas (USA). In the continuity of these works, Liu et al. discussed the positive role of self-efficacy in the relationship between age, social isolation and poorer nutritional literacy in rural China, which is associated with a higher number of chronic diseases. Then, in the suburban area of Berlin in Germany, a contribution by Darkhani underlined the positive role of women in the development of alternative food networks to favor environmentally sustainable modes of production, certified food quality, food safety consciousness, and health and nutrition concerns. In addition, Brukalo et al. highlighted how public procurement of fatty products for Polish educational units can be improved to promote children's health. Hence, standardized guidelines are required to promote healthier food choices, encourage sustainable diets, and ultimately improve the overall health and wellbeing of children. Finally, in the context of the current ecological crisis, specifically the increasing number of natural disasters in Indonesia, a clinical trial conducted by Fatmah demonstrated that *Api-api* mangrove, an abundant plant species in coastal areas of the country, consumed as sword bean snack bars constitutes a viable and efficient substitute for emergency food provisions, particularly in disaster-stricken communities.

Accordingly, based on these fruitful contributions, the SDG 10 provides new insights to identify at-risk subgroups for nutrition-related diseases along the global urban transition. Children under five years, older adults and socially excluded populations are all subgroups overexposed to stunting, wasting or overweight, depending on the current stage of urban transition in their respective countries. This Research Topic innovatively presents a clear overview of the ongoing nutritional transition associated with the globalized urbanization process. Moreover, the SDG 10 describes multiple local, national and regional alternatives worldwide to address the public health nutritional issues stemming from this urban transition. Alternative food networks, such as innovative agro-food systems where women can play a major role in Germany; school gardening and nutrition education in Texas (USA); or multilevel food intervention programs based on sustainable local production, food safety and food diversity in Nigeria, constitute a small sample from this large landscape of adaptive strategies to allow healthy population and individual trajectories in growing urban areas worldwide.
